# (*Z*)-5-Fluoro-3-[(1*H*-pyrrol-2-yl)methyl­ene]indolin-2-one

**DOI:** 10.1107/S1600536808040178

**Published:** 2008-12-03

**Authors:** Hongming Zhang, Haribabu Ankati, Shashidhar Kumar Akubathini, Ed Biehl

**Affiliations:** a Department of Chemistry, Southern Methodist University, Dallas, TX 75275, USA

## Abstract

The title compound, C_13_H_9_FN_2_O, a potential neuroprotective agent, consists of an indolinone and a pyrrolyl unit [dihedral angle between the ring planes = 4.9 (1)°]. An intra­molecular hydrogen bond between the carbonyl O atom and the NH group of pyrrole correlates with the *Z* arrangement of the substituents at the C=C bond. In the crystal, inversion dimers occur, linked by pairs of N—H⋯O bonds.

## Related literature

For 3-substituted indole-2-one derivatives tested as protein kinase inhibitors, see: Sun *et al.* (2003 ▶). For derivatives with anti­tumor activity, see: Andreani *et al.* (2006 ▶). For derivatives with neuroprotective properties, see: Balderamos *et al.* (2008 ▶); Johnson *et al.* (2005 ▶). For related structures, see: Ali *et al.* (2008 ▶); De (2008 ▶); Zhang *et al.* (2008 ▶).
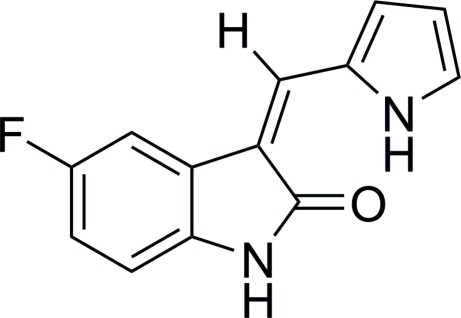


## Experimental

### 

#### Crystal data

C_13_H_9_FN_2_O
*M* *_r_* = 228.22
Monoclinic, 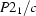

*a* = 7.6093 (5) Å
*b* = 6.1270 (4) Å
*c* = 22.8912 (16) Å
β = 91.390 (1)°
*V* = 1066.92 (12) Å^3^
*Z* = 4
Mo *K*α radiation
μ = 0.10 mm^−1^
*T* = 293 (2) K
0.35 × 0.24 × 0.08 mm

#### Data collection

Bruker APEX diffractometer
Absorption correction: multi-scan (*SADABS*; Sheldrick, 1996 ▶) *T*_min_ = 0.964, *T*_max_ = 0.992
12282 measured reflections
2552 independent reflections
2167 reflections with *I* > 2σ(*I*)
*R*_int_ = 0.022

#### Refinement

*R*[*F* ^2^ > 2σ(*F* ^2^)] = 0.054
*wR*(*F* ^2^) = 0.137
*S* = 1.10
2552 reflections
154 parameters
H-atom parameters constrained
Δρ_max_ = 0.23 e Å^−3^
Δρ_min_ = −0.22 e Å^−3^

### 

Data collection: *SMART* (Bruker, 1997 ▶); cell refinement: *SAINT* (Bruker, 1997 ▶); data reduction: *SAINT*; program(s) used to solve structure: *SHELXS97* (Sheldrick, 2008 ▶); program(s) used to refine structure: *SHELXL97* (Sheldrick, 2008 ▶); molecular graphics: *SHELXTL* (Sheldrick, 2008 ▶); software used to prepare material for publication: *SHELXTL* and *publCIF* (Westrip, 2008 ▶).

## Supplementary Material

Crystal structure: contains datablocks I, global. DOI: 10.1107/S1600536808040178/si2132sup1.cif

Structure factors: contains datablocks I. DOI: 10.1107/S1600536808040178/si2132Isup2.hkl

Additional supplementary materials:  crystallographic information; 3D view; checkCIF report

## Figures and Tables

**Table 1:** Hydrogen-bond geometry (Å, °)

*D*—H⋯*A*	*D*—H	H⋯*A*	*D*⋯*A*	*D*—H⋯*A*
N1—H1⋯O2^i^	0.86	2.03	2.8711 (17)	166
N11—H11⋯O2	0.86	1.94	2.6977 (19)	147

Symmetry code: (i) 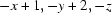.

**Fig. 1.**
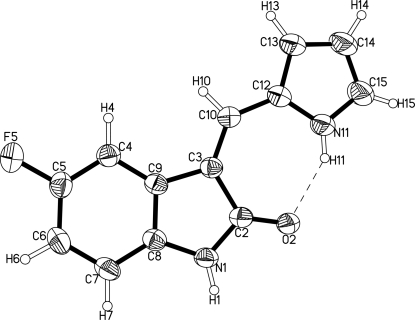
: A view of one of the independent molecules with displacement ellipsoids drawn at the 40% probability level. Dash lines indicate the intramolecular hydrogen bond. H atoms are presented as open circles with arbitrary radii.

**Fig. 2.**
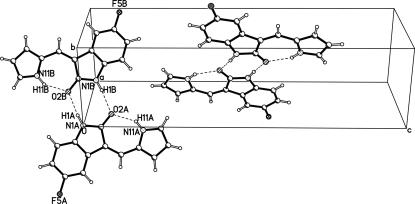
: A unit cell packing view of the title compound. Dash lines indicate the intra- and intermolecular hydrogen bonds. H atoms are presented as open circles with arbitrary radii.

## supplementary crystallographic information

### Comment

3-Substituted indoline-2-ones are a variety of pharmacologically important compounds such as protein kinase inhibitors (Sun *et al.*, 2003), antitumor (Andreani *et al.*, 2006) and neuroprotective properties (Balderamos *et al.*, 2008; Johnson *et al.*, 2005). We have designed, synthesized and crystallized several 3-substituted indoline-2-one derivatives to study their neuroprotective properties. In order to study on the relationship between the activity of 3-substituted indoline-2-ones and the importance of halogenated substituent at the 5-postion, the fluoro derivative was synthesized and its crystal structure is reported here. An intramolecular hydrogen bond was found between the N—H of pyrrole and the carbonyl O and a hepta cyclic membered ring was formed (Table 1) (Fig 1). Unlike the *E* arrangement of the chloro derivative (Zhang, *et al.*, 2008), thanks to the intramolecular H bond, the title compound adopted a *Z* conformation (Fig 1). Compared to the bond length C – Cl 1.736 (5) Å, the C – F is 1.364 (2) Å in the current structure, similar to other indolin-2-one compounds (Ali, *et al.*, 2008; De, 2008; Zhang, *et al.*, 2008;) which contain intermolecular N—H···O hydrogen bonds. The H-bonds link two inverted molecules, forming an octa cyclic membered ring, and a dimer is constructed (Table 1) (Fig 2).

### Experimental

The title compound was synthesized by the condensation of pyrrole-2-carboxaldehyde (1 mmol) with 5-fluoro-oxindole (1 mmol) in ethanol (10 ml) in the presence of catalytic amount of piperidine (0.1 mmol). After refluxing for 3 hr, the reaction mixture was left to stand for overnight. The resulting crude solid was filtered, washed with cold ethanol (10 ml) and dried. Orange red single crystals of the compound suitable for *x*-ray structure determination were recrystallized from ethanol.

### Refinement

All H atom were placed in calculated positions and included in the final cycles of refinement using a riding model, with distances N–H = 0.86 Å and C–H = 0.93 Å, and displacement parameters *U*_iso_(H) = 1.2*U*_eq_(N,C).

### Crystal data

**Table:**

C_13_H_9_FN_2_O	*F*(000) = 472
*M**_r_* = 228.22	*D*_x_ = 1.421 Mg m^−^^3^
Monoclinic, *P*2_1_/*c*	Mo *K*α radiation, λ = 0.71073 Å
*a* = 7.6093 (5) Å	Cell parameters from 3595 reflections
*b* = 6.1270 (4) Å	θ = 2.7–25.4°
*c* = 22.8912 (16) Å	µ = 0.10 mm^−^^1^
β = 91.390 (1)°	*T* = 293 K
*V* = 1066.92 (12) Å^3^	Plates, orange
*Z* = 4	0.35 × 0.24 × 0.08 mm

### Data collection

**Table:**

Bruker APEX diffractometer	2552 independent reflections
Radiation source: fine-focus sealed tube	2167 reflections with *I* > 2σ(*I*)
graphite	*R*_int_ = 0.022
Detector resolution: 83.33 pixels mm^-1^	θ_max_ = 28.3°, θ_min_ = 1.8°
φ and ω scans	*h* = −9→9
Absorption correction: multi-scan (*SADABS*; Sheldrick, 1996)	*k* = −8→8
*T*_min_ = 0.964, *T*_max_ = 0.992	*l* = −29→29
12282 measured reflections	

### Refinement

**Table:**

Refinement on *F*^2^	Primary atom site location: structure-invariant direct methods
Least-squares matrix: full	Secondary atom site location: difference Fourier map
*R*[*F*^2^ > 2σ(*F*^2^)] = 0.054	Hydrogen site location: inferred from neighbouring sites
*wR*(*F*^2^) = 0.137	H-atom parameters constrained
*S* = 1.10	*w* = 1/[σ^2^(*F*_o_^2^) + (0.0587*P*)^2^ + 0.3105*P*] where *P* = (*F*_o_^2^ + 2*F*_c_^2^)/3
2552 reflections	(Δ/σ)_max_ < 0.001
154 parameters	Δρ_max_ = 0.23 e Å^−^^3^
0 restraints	Δρ_min_ = −0.22 e Å^−^^3^

### Special details

**Table:**

Geometry. All e.s.d.'s (except the e.s.d. in the dihedral angle between two l.s. planes) are estimated using the full covariance matrix. The cell e.s.d.'s are taken into account individually in the estimation of e.s.d.'s in distances, angles and torsion angles; correlations between e.s.d.'s in cell parameters are only used when they are defined by crystal symmetry. An approximate (isotropic) treatment of cell e.s.d.'s is used for estimating e.s.d.'s involving l.s. planes.
Refinement. Refinement of *F*^2^ against ALL reflections. The weighted *R*-factor *wR* and goodness of fit *S* are based on *F*^2^, conventional *R*-factors *R* are based on *F*, with *F* set to zero for negative *F*^2^. The threshold expression of *F*^2^ > σ(*F*^2^) is used only for calculating *R*-factors(gt) *etc*. and is not relevant to the choice of reflections for refinement. *R*-factors based on *F*^2^ are statistically about twice as large as those based on *F*, and *R*- factors based on ALL data will be even larger.

### Fractional atomic coordinates and isotropic or equivalent isotropic displacement parameters (Å^2^)

**Table:**

	*x*	*y*	*z*	*U*_iso_*/*U*_eq_	
N1	0.36941 (18)	0.7704 (2)	−0.02026 (6)	0.0462 (3)	
H1	0.4156	0.8783	−0.0384	0.055*	
C2	0.3608 (2)	0.7558 (3)	0.03865 (7)	0.0422 (4)	
O2	0.41973 (16)	0.89761 (19)	0.07266 (5)	0.0515 (3)	
C3	0.27094 (19)	0.5468 (2)	0.05187 (7)	0.0395 (3)	
C4	0.1488 (2)	0.2559 (3)	−0.02156 (7)	0.0457 (4)	
H4	0.1059	0.1591	0.0060	0.055*	
C5	0.1325 (2)	0.2144 (3)	−0.08055 (8)	0.0503 (4)	
F5	0.04936 (17)	0.0275 (2)	−0.09798 (5)	0.0727 (4)	
C6	0.1934 (2)	0.3515 (3)	−0.12313 (7)	0.0536 (4)	
H6	0.1788	0.3157	−0.1624	0.064*	
C7	0.2772 (2)	0.5443 (3)	−0.10659 (7)	0.0511 (4)	
H7	0.3204	0.6399	−0.1343	0.061*	
C8	0.2942 (2)	0.5888 (3)	−0.04789 (7)	0.0421 (4)	
C9	0.23179 (19)	0.4481 (2)	−0.00504 (6)	0.0395 (3)	
C10	0.2305 (2)	0.4604 (3)	0.10429 (7)	0.0448 (4)	
H10	0.1771	0.3241	0.1019	0.054*	
N11	0.3347 (2)	0.7320 (3)	0.17745 (6)	0.0587 (4)	
H11	0.3819	0.8215	0.1535	0.070*	
C12	0.2548 (2)	0.5398 (3)	0.16222 (7)	0.0488 (4)	
C13	0.1974 (3)	0.4470 (4)	0.21346 (8)	0.0667 (6)	
H13	0.1390	0.3144	0.2167	0.080*	
C14	0.2423 (4)	0.5862 (4)	0.25906 (9)	0.0792 (7)	
H14	0.2184	0.5654	0.2983	0.095*	
C15	0.3282 (4)	0.7598 (4)	0.23562 (9)	0.0756 (7)	
H15	0.3746	0.8777	0.2564	0.091*	

### Atomic displacement parameters (Å^2^)

**Table:**

	*U*^11^	*U*^22^	*U*^33^	*U*^12^	*U*^13^	*U*^23^
N1	0.0597 (8)	0.0390 (7)	0.0402 (7)	−0.0043 (6)	0.0079 (6)	0.0063 (5)
C2	0.0462 (8)	0.0393 (8)	0.0414 (8)	0.0013 (6)	0.0063 (6)	0.0043 (6)
O2	0.0673 (8)	0.0445 (6)	0.0431 (6)	−0.0124 (5)	0.0075 (5)	−0.0007 (5)
C3	0.0408 (7)	0.0384 (8)	0.0395 (8)	0.0016 (6)	0.0050 (6)	0.0011 (6)
C4	0.0496 (9)	0.0423 (9)	0.0453 (9)	−0.0009 (7)	0.0042 (7)	0.0017 (7)
C5	0.0562 (10)	0.0442 (9)	0.0505 (10)	0.0006 (7)	−0.0014 (7)	−0.0068 (7)
F5	0.0964 (9)	0.0608 (7)	0.0608 (7)	−0.0194 (6)	−0.0027 (6)	−0.0137 (6)
C6	0.0669 (11)	0.0565 (10)	0.0374 (8)	0.0071 (9)	−0.0002 (7)	−0.0055 (7)
C7	0.0663 (11)	0.0503 (10)	0.0370 (8)	0.0057 (8)	0.0066 (7)	0.0063 (7)
C8	0.0465 (8)	0.0390 (8)	0.0409 (8)	0.0055 (6)	0.0036 (6)	0.0038 (6)
C9	0.0411 (7)	0.0401 (8)	0.0374 (8)	0.0053 (6)	0.0047 (6)	0.0027 (6)
C10	0.0473 (8)	0.0448 (9)	0.0425 (9)	−0.0051 (7)	0.0041 (6)	0.0037 (7)
N11	0.0806 (11)	0.0554 (9)	0.0404 (8)	−0.0147 (8)	0.0106 (7)	0.0001 (6)
C12	0.0545 (9)	0.0523 (10)	0.0398 (9)	−0.0033 (7)	0.0046 (7)	0.0045 (7)
C13	0.0903 (15)	0.0664 (13)	0.0436 (10)	−0.0170 (11)	0.0087 (9)	0.0076 (9)
C14	0.124 (2)	0.0762 (15)	0.0380 (10)	−0.0175 (14)	0.0131 (11)	0.0031 (9)
C15	0.1179 (18)	0.0673 (13)	0.0420 (10)	−0.0167 (13)	0.0082 (11)	−0.0050 (9)

### Geometric parameters (Å, °)

**Table:**

N1—C2	1.355 (2)	C7—C8	1.374 (2)
N1—C8	1.396 (2)	C7—H7	0.9300
N1—H1	0.8600	C8—C9	1.397 (2)
C2—O2	1.2429 (19)	C10—C12	1.421 (2)
C2—C3	1.486 (2)	C10—H10	0.9300
C3—C10	1.354 (2)	N11—C15	1.344 (2)
C3—C9	1.460 (2)	N11—C12	1.367 (2)
C4—C5	1.377 (2)	N11—H11	0.8600
C4—C9	1.385 (2)	C12—C13	1.384 (2)
C4—H4	0.9300	C13—C14	1.385 (3)
C5—F5	1.364 (2)	C13—H13	0.9300
C5—C6	1.376 (3)	C14—C15	1.365 (3)
C6—C7	1.390 (3)	C14—H14	0.9300
C6—H6	0.9300	C15—H15	0.9300
			
C2—N1—C8	111.66 (13)	N1—C8—C9	108.45 (14)
C2—N1—H1	124.2	C4—C9—C8	119.57 (14)
C8—N1—H1	124.2	C4—C9—C3	132.66 (14)
O2—C2—N1	123.50 (14)	C8—C9—C3	107.77 (14)
O2—C2—C3	129.46 (14)	C3—C10—C12	131.79 (16)
N1—C2—C3	107.04 (14)	C3—C10—H10	114.1
C10—C3—C9	125.66 (15)	C12—C10—H10	114.1
C10—C3—C2	129.26 (15)	C15—N11—C12	109.57 (16)
C9—C3—C2	105.08 (13)	C15—N11—H11	125.2
C5—C4—C9	117.01 (15)	C12—N11—H11	125.2
C5—C4—H4	121.5	N11—C12—C13	106.60 (16)
C9—C4—H4	121.5	N11—C12—C10	125.47 (15)
F5—C5—C6	117.86 (15)	C13—C12—C10	127.88 (17)
F5—C5—C4	118.16 (16)	C12—C13—C14	107.97 (19)
C6—C5—C4	123.97 (16)	C12—C13—H13	126.0
C5—C6—C7	119.06 (15)	C14—C13—H13	126.0
C5—C6—H6	120.5	C15—C14—C13	107.19 (18)
C7—C6—H6	120.5	C15—C14—H14	126.4
C8—C7—C6	117.81 (15)	C13—C14—H14	126.4
C8—C7—H7	121.1	N11—C15—C14	108.66 (19)
C6—C7—H7	121.1	N11—C15—H15	125.7
C7—C8—N1	128.97 (15)	C14—C15—H15	125.7
C7—C8—C9	122.58 (15)		

### Hydrogen-bond geometry (Å, °)

**Table:**

*D*—H···*A*	*D*—H	H···*A*	*D*···*A*	*D*—H···*A*
N1—H1···O2^i^	0.86	2.03	2.8711 (17)	166
N11—H11···O2	0.86	1.94	2.6977 (19)	147

Symmetry codes: (i) −*x*+1, −*y*+2, −*z*.
